# Case Report: Concordance of cerebrospinal fluid-derived circulating tumor DNA with tumor tissue in spinal glioblastoma associated with Lynch syndrome

**DOI:** 10.3389/fonc.2026.1914228

**Published:** 2026-09-09

**Authors:** Carlen A. Yuen, David O. Kamson, Changrui Xiao, William H. Yong, Jennifer E. Soun, Mark Linskey, Michelle Paff, Caressa Hui, Alexander M. Lopez, Yael Freiberg, Hansen Bow

**Affiliations:** 1 Department of Neurology, Division of Neuro-oncology, University of California, Irvine, Irvine, CA, United States; 2 Chao Family Comprehensive Cancer Center, University of California, Irvine, Irvine, CA, United States; 3 The Sidney Kimmel Comprehensive Cancer Center, Johns Hopkins University, Baltimore, MD, United States; 4 Department of Neurology, School of Medicine, Johns Hopkins University, Baltimore, MD, United States; 5 Department of Neurology, Division of Neuro-Genetics, Chao Family Comprehensive Cancer Center, University of California, Irvine, Irvine, CA, United States; 6 Department of Pathology, Division of Neuropathology, University of California, Irvine, Irvine, CA, United States; 7 Department of Radiology, Division of Neuroradiology, University of California, Irvine, Irvine, CA, United States; 8 Department of Neurosurgery, University of California, Irvine, Irvine, CA, United States; 9 Department of Radiation Oncology, University of California, Irvine, Irvine, CA, United States; 10 Department of Pediatrics, Division of Genetic and Genomic Medicine, University of California, Irvine, Irvine, CA, United States

**Keywords:** Belay, cell-free DNA, circulating tumor (ctDNA), ctDNA, glioblastoma, immunotherapy, Lynch syndrome, spinal glioblastoma

## Abstract

**Background:**

Spinal glioblastoma (GBM) is an exceptionally rare entity and poses significant diagnostic challenges due to a substantial risk of neurological morbidity with surgical biopsy. Tissue-based analyses are frequently limited by sampling constraints and tumor heterogeneity. Conventional diagnostic modalities, including MRI and cerebrospinal fluid (CSF) cytology, often lack specificity and sensitivity. Commercially available CSF liquid biopsy platforms offer a minimally invasive approach for detecting tumor-derived cell-free DNA (cfDNA) to enable genomic profiling.

**Methods:**

We report a case of a 37-year-old male with spinal GBM who underwent prospective CSF cfDNA analysis using Belay Summit™ and GTC Liquid Trace™, yielding a provisional diagnosis of GBM and raising suspicion for Lynch syndrome.

**Results:**

Subsequent tissue-based molecular profiling with Tempus™ confirmed the diagnosis of GBM, while germline testing via Ambry Genetics™ established Lynch syndrome.

**Conclusion:**

Based on the findings of mismatch repair deficiency and Lynch syndrome, the decision to proceed with immunotherapy was made. CSF cfDNA is a promising ancillary tool for guiding therapy in difficult-to-biopsy cases.

## Introduction

Genomic profiling is integral to glioblastoma (GBM) management and enables molecular classification and therapeutic stratification. Following the 2021 World Health Organization (WHO) classification of central nervous system (CNS) tumors, GBM diagnosis increasingly relies upon sequencing-defined molecular alterations, including *IDH* mutation status, *EGFR* amplification, *CDKN2A/B* homozygous deletion, *TERT* promoter mutation, and combined whole chromosome 7 gain/10 loss, which complement or supersede histopathologic criteria (1). Beyond *MGMT* promoter methylation status, additional predictive biomarkers, including microsatellite instability (MSI) and tumor mutational burden (TMB), carry clinically actionable relevance.

Elevated TMB and MSI-high status are associated with enhanced responsiveness to immune checkpoint blockade and underpin the tumor-agnostic FDA approvals of these agents, including nivolumab and pembrolizumab. Identification of mismatch repair (MMR) deficiency is therefore critical for guiding immunotherapeutic strategies in select GBM patients.

In the landmark report of two pediatric siblings with constitutional mismatch repair deficiency (CMMRD) and recurrent multifocal GBM, durable response to single-agent nivolumab was achieved following progression with standard therapy (2). This outcome markedly diverges from the typical aggressive clinical course of recurrent GBM patients. CMMRD is characterized by loss of mismatch repair function due to biallelic pathogenic variants of DNA mismatch repair genes *MLH1*, *MSH2*, *MSH6*, or *PMS2*, resulting in high TMB and markedly elevated neoantigen burden (2).

Lynch syndrome is allelic with CMMRD, but affected individuals are heterozygous for germline variants, causing genetic susceptibility to malignancies including GBM (3). Although Lynch syndrome-associated gliomas are recognized, the clinical behavior and survival outcomes are poorly characterized with fewer than five cases reported to date (**Table 1**). Spinal GBM constitutes an exceptionally rare subset within this already uncommon disease entity.

**Table 1:** Lynch syndrome-associated glioblastoma treated with PD-1 blockade.

Case	Age/Sex	Initial treatment	Progressive Disease	PD-1 blockade	Outcome
Present Case	36/M	Cyclophosphamide →TMZ x3 days → NTR → radiation therapy → pembrolizumab	–	pembrolizumab	ongoing
Sherman et al. (15)	57/F	Left occipital resection → RT (60 Gy) + TMZ, followed by TTF. Right frontal recurrence with resection → RT + nivolumab	–	nivolumab concurrently with radiation	5 years out from initial diagnosis
Anghileri et al. (16)	33/F	Frontal resection → radiotherapy, and TMZ → re-recurrence + GTR with surgery → nivolumab	15 months	second surgery and treatment with nivolumab	Receiving nivolumab 84 months after surgery
Cabezas-Camarero et al. (17)	34/M	Biopsy → TMZ × 12 cycles → partial resection→ RT (60 Gy) → fotemustine → bevacizumab × 27 cycles → WandS × 12 m → bevacizumab × 13 cycles → tumor progression and biopsy → nivolumab → irinotecan + bevacizumab	–	Third Surgery and compassionate authorization of nivolumab	Comfort care, 7 years from diagnosis

NTR, near total resection; RT, radiation therapy; TMZ, temozolomide; TTF, tumor-treating fields; GTR, gross total resection.

Liquid biopsy has emerged as a minimally invasive approach for both diagnostic and real-time longitudinal monitoring of tumor burden and molecular status through analysis of circulating tumor DNA (ctDNA) that is shed by the tumor (4). While plasma-based and serum-based ctDNA assays are well established in systemic malignancies, these assays have limited utility in CNS tumors due to the low contribution of tumor DNA into the peripheral blood due to the blood–brain barrier. However, CSF provides direct access to the CNS tumor microenvironment, enabling more sensitive detection of tumor-specific genomic alterations.

Although integration of CSF-derived ctDNA into routine clinical workflows for CNS tumors remains investigational, the field is rapidly advancing. CSF platforms, including Belay Summit™ and GTC Liquid Trace™, enable genomic and epigenomic profiling from low-input CSF tumor-derived DNA. These assays also enable the assessment of MSI and TMB. However, data evaluating the concordance between CSF-derived ctDNA and matched tumor tissue remain limited (5).

We present a case of provisionally diagnosed Lynch-associated spinal GBM based on CSF ctDNA analysis with Belay Summit™ and GTC Liquid Trace™ diagnostic testing. Tempus™ confirmed GBM, and Ambry™ germline testing confirmed Lynch syndrome. This case underscores the clinical utility of CSF liquid biopsy for CNS tumor profiling and partial concordance to matched tissue-based next-generation sequencing.

## Methods

Patient clinical data were retrospectively collected during the course of routine clinical care and subsequently reviewed for this case report. CSF was collected for ctDNA analysis using the Belay Summit™ and GTC Liquid Trace™ assays. Tissue-based molecular profiling was performed using the Tempus™ next-generation sequencing platform. These clinically validated assays assessed single-nucleotide variants, insertions/deletions, copy number alterations, gene fusions, and TMB in cancer-associated genes according to the manufacturers’ validated protocols. Sequencing results were interpreted in the context of our patient’s clinical history, radiographic findings, and histopathologic features. Confirmatory germline testing was performed using Ambry™ multigene panel testing.

## Case description

**Figure 1:**
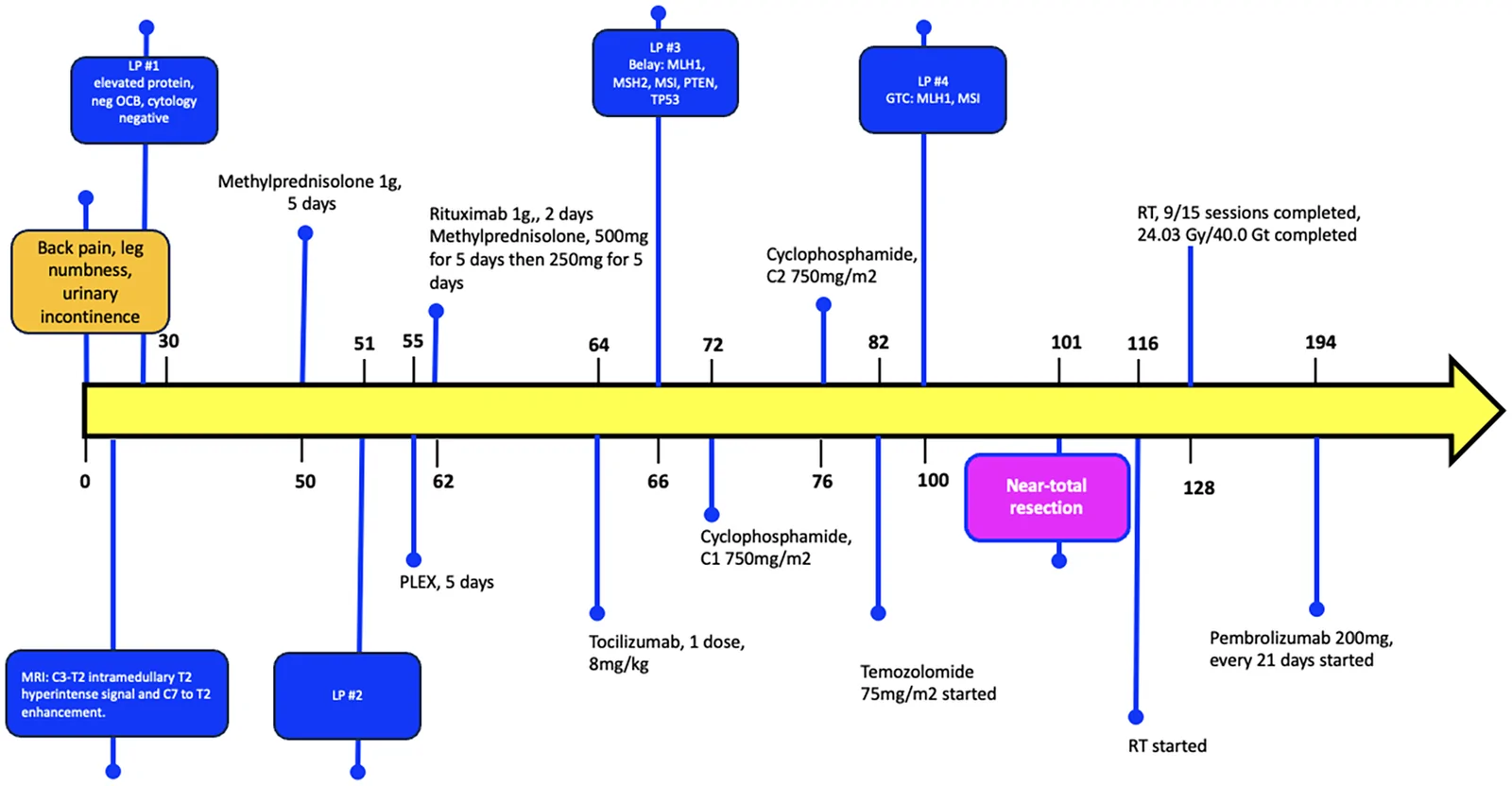
Patient disease course.

**Figure 2:**
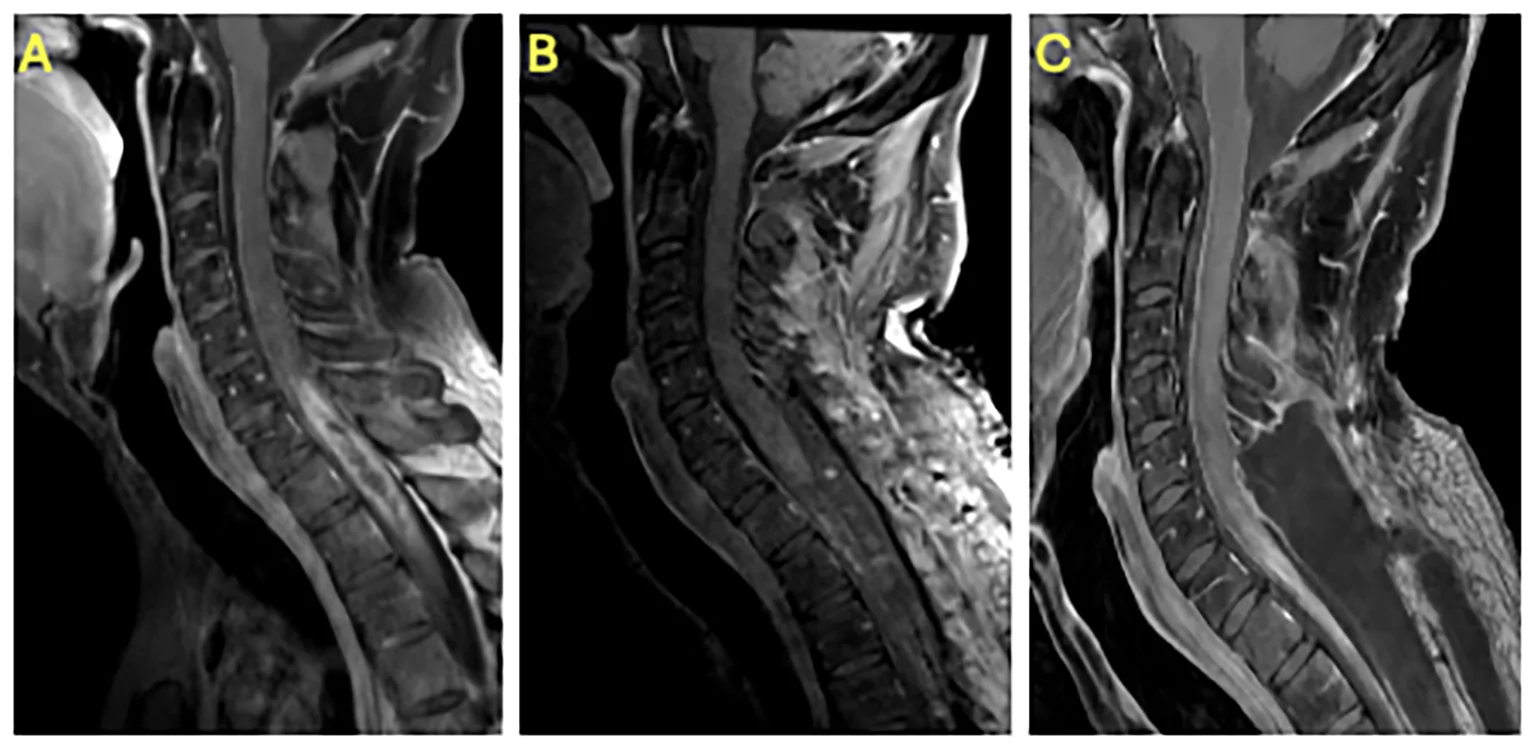
Postcontrast sagittal T1 fat-suppressed sequences demonstrating the tumor **(A)** pretreatment, **(B)** postoperative, and **(C)** post-radiation. **(A)** Preoperative: heterogeneously enhancing intramedullary tumor spanning the C6-T2 spinal cord. **(B)** Postoperative: C6-T4 laminectomies and expansion duroplasty for microsurgical resection of the intramedullary tumor. Residual mild intramedullary enhancement. **(C)** Post-radiation: Increased intramedullary enhancement at the treatment bed. New pseudomeningocele in the posterior soft tissues.

A 36-year-old man with no significant past medical history presented to an outside hospital with progressive bilateral lower-extremity weakness, a sensory level below the chest, and urinary incontinence (**Figure 1**). MRI of the complete spine demonstrated an enhancing intramedullary lesion spanning C6-T2 (**Figure 2**). His family history was notable for maternally reported Lynch syndrome.

Within 2 weeks, he deteriorated to flaccid paraplegia with bowel and bladder dysfunction. Empiric treatment with plasmapheresis, intravenous methylprednisolone, rituximab, and tocilizumab yielded no clinical improvement. CSF studies, including cytology, showed negative results (**Table 2**). Belay Summit™ 2.0 CSF ctDNA detected a *PTEN* mutation (variant allele frequency (VAF) 10.6%) and MMR alterations (*MLH1* VAF 54.1%, *MSH2* VAF 2.2%) with MSI, raising suspicion for GBM in the setting of Lynch syndrome (**Table 3**). Temozolomide (TMZ) (150 mg/m²) was initiated but discontinued after 3 days due to constipation and abdominal distention.

**Table 2:** Patient’s CSF studies.

CSF study (#)	1 (D0)	2 (D51)	3 (D66)	4 (D100)
OP (cm H20)	Noted as normal	Not obtained	Not obtained	22
ml	23	10	unknown	26
RBC (cumm)	0	33	3	56
WBC (cumm)	4	11	5	1
Lymphocytes (%)	55	57	81	31
Neutrophils (%)	0	22	0	12
Monocytes (%)	45	21	19	58
Glucose (mg/dL)	106	45	65	63
Protein (mg/dL)	61	81	97	58
Culture	negative	negative		negative
Meningitis/encephalitis panel (PCR)^1^	–	–	–	negative
ACE, quantitative (U/L)	<5	6	5	–
HSV 1/2	Not detected	Not detected	–	–
VDRL, qualitative	Non-reactive	Non- reactive	–	–
WNV IgM, qualitative, EIA	<0.90	–	–	–
Coccidioides	–	–	–	No antibodies detected
Cryptococcus Antigen	–	–	–	negative
IgG (mg/dL)	5.4	–	–	negative
OCG	absent	absent	–	negative
Encephalitis/Autoimmune/Paraneoplastic Panel^2^	–	negative	negative	negative
Cytology	negative	negative	–	negative
Flow Cytometry	–	–	–	negative

-, not obtained. D, day; OP, opening pressure; PCR, polymerase chain reaction; ACE, angiotensin-converting enzyme; HSV, herpes simplex virus; VDRL, venereal disease research laboratory; WNV, West Nile Virus; IgG, immunoglobulin G; OCG, oligoclonal bands.

^1^ Escherichia coli K1, Haemophilus influenzae, Listeria monocytogenes, Neisseria meningitidis, Streptococcus agalactiae, Streptococcus pneumoniae, Cytomegalovirus, Enterovirus, Herpes simplex virus 1, Herpes simplex virus 2, Human herpesvirus 6, Human parechovirus, Varicella zoster virus, Cryptococcus neoformans/gattii.

^2^ AGNA-1, Amphiphysin Ab, ANNA-1, ANNA-2, ANNA-3, CRMP-5-IgG, PCA-1, PCA-2, PCA-Tr, GAD65 Ab Assay, NMDA-R Ab CBA, AMPA-R Ab CBA, GABA-B-R Ab CBA, LGI1-IgG CBA, CASPR2-IgG CBA, mGluR1 Ab IFA, GFAP IFA, NIF IFA, Neurochondrin IFA, Septin-7 IFA, DPPX Ab CBA, IgLON5 CBA, PDE10A Ab IFA, TRIM46 Ab IFA.

**Table 3:** Comparison of patient’s molecular analyses.

Gene	CSF				Tumor tissue		Saliva
	Genomic testing cooperative liquid trace		Belay summit 2.0		Tempus		Ambry genetics
	Genomic variant	VAF (%)	Genomic variant	VAF (%)	Genomic variant	VAF (%)	
Pathogenic – germline implications							
BRCA1	Not detected		p.E23fs c.68_69del	3.8	p.E23fs - LOF	22.4	Not detected
**MLH1**	**c.589-2A>G**	** 43.56**	**c.589-2A>G**	**54.1**	**c.589-2A>G Splice region variant - LOF**	**71.7**	**c.589-2A->G** (confirmed germline)
MSH2	Not detected		p.I883fs c.2647du	2.2	p.I883fs - LOF	13.5	Not detected
Glioblastoma (somatic)							
MGMTp			Unmethylated		Unmethylated		
CDKN2A	Not detected		CDKN2A p.R58* c.172C>T	4.4	p.R58* Stop gain - LOF	52.7	
EGFR	Low level gain in autosomal chromosomes		Not detected		Not detected		
PTEN	Not detected		p.N323fs c.968del	10.6	p.N323fs - LOF	30.8	
TP53	Not detected		p.G245S c.733G>A	1.9			
Pathogenic - actionable							
MTOR	Not detected		Not detected		p.L2431P Missense variant - GOF	6.6	
NF1	Not detected		Not detected		p.I679fs - LOF	53.7	
PIK3R1	Not detected		c.1746-2A>G	3.9	c.1746-2A>G Splice variant - LOF	11.8	
Pathogenic - non-actionable							
AJUBA	Not tested		Not tested		p.A244fs - LOF	25.6	
ARID1A	Not detected		p.Y815* c.2445_2446d	7.6	p.Y815fs - LOF	26.2	
BCORL1	Not detected		P1681fs***		p.1755fs - LOF	61.4	
KMT2D	Not detected		Not detected		p.R2915* Stop gain - LOF	32.2	
MRE11	Not detected		p.T481fs c.1441dup	6.7	p.T481fs - LOF	28.3	
NOTCH2	c.74C>A	22.22	Not detected				
TCF7L2	Not tested		Not detected		p.C469fs - GOF	31.7	
Other actionable							
TMB	Low (1mut/Mb)		Not detected		13.7mut/MB (92nd %ile)		
MSI	Not tested		High		High		

The * and *** notations are a part of the allele nomenclature.

The bold values indicate concordance among the CSF, tissue, and saliva assays.

Upon transfer to our institution, examination revealed American Spinal Injury Association (ASIA) A T1 complete paraplegia with preserved hand function (partial C7-C8 involvement). Repeat CSF ctDNA with GTC Liquid Trace™ confirmed *MLH1* alteration with low TMB and low-level *EGFR* gain. Given the discordant results from the Belay Summit™ and GTC Liquid Trace™ platforms, which were obtained at different time points in the disease course and that paraplegia was already established, a tissue-based diagnosis was pursued.

**Figure 3:**
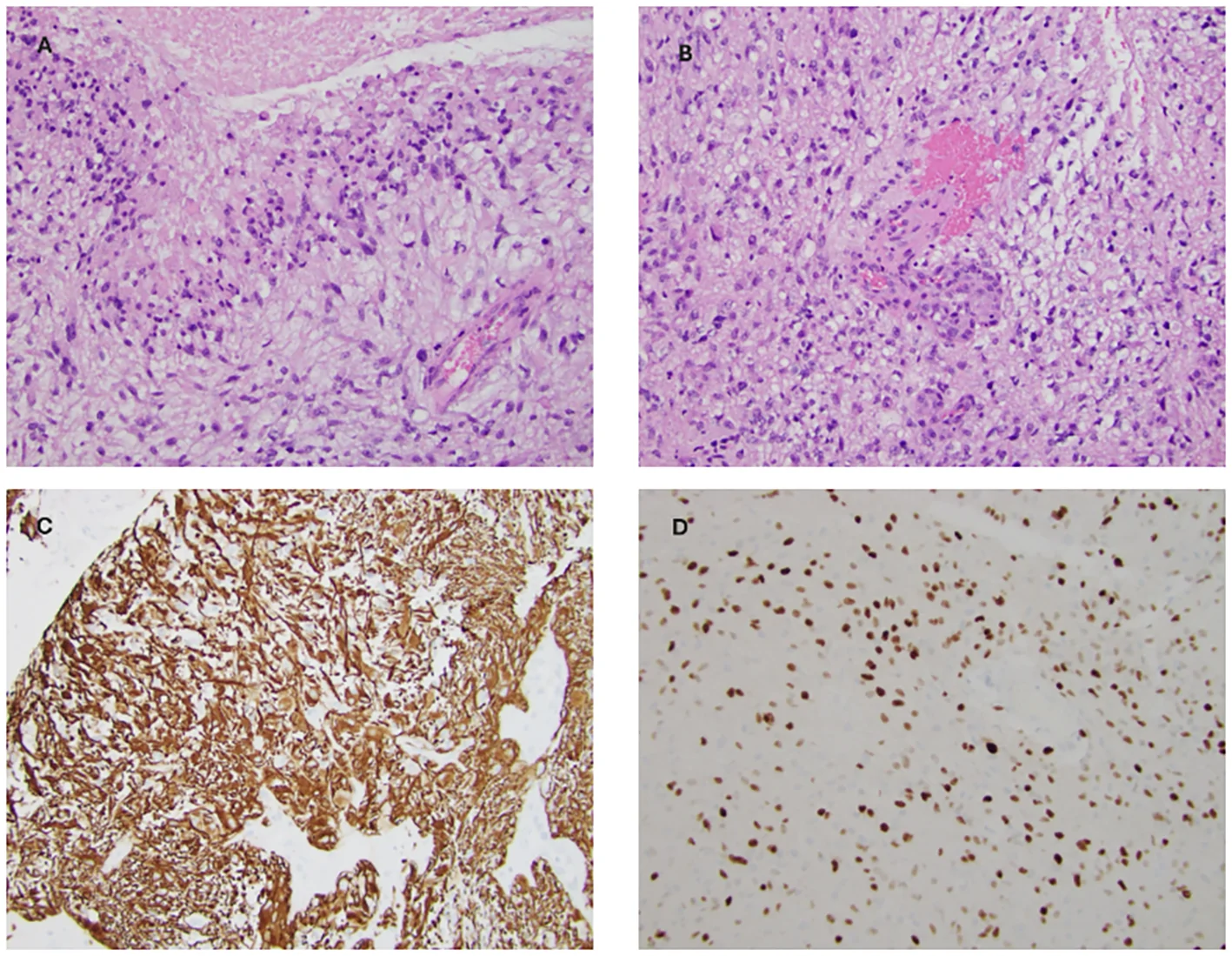
**(A)** H&E-fibrillary astrocytes with palisading necrosis (200×). **(B)** H&E-microvascular proliferation (200×). **(C)** GFAP-fibrillary and gemistocytic astrocytes (200×). **(D)** Olig2-extensive nuclear immunopositivity (400×).

The patient underwent C6-T3 laminectomy with subtotal resection of the T1–3 intramedullary tumor and expansion duroplasty, with superior resection limited to preserving hand function. Postoperatively, neurological status was unchanged. Histopathology demonstrated a hypercellular astrocytic neoplasm with palisading necrosis and microvascular proliferation, immunopositive for Olig2, ATRX, and GFAP and negative for *SOX10*, *EMA*, *IDH1* R132H, and *BRAF V600E*. Ki-67 showed a score of 5%-10% (**Figure 3**).

Tumor next-generation sequencing with Tempus™ confirmed MSI with *MLH1* c.589-2A>G splice-site mutation (VAF 71.7%), *PTEN* and *MSH2* frameshift loss-of-function variants, and unmethylated *MGMT*. Additional alterations included *BCORL1*, *NF1*, and *CDKN2A* loss-of-function mutations, and alterations in *KMT2D, MRE11, ARID1A, AJUBA*, and *TCF7L2*. No canonical *IDH1/2, H3K27M*, and *TERT* promoter mutations or *EGFR* amplification was identified. TMB was high in contrast to GTC Liquid Trace™ findings. The tumor was classified as Glioblastoma, IDH-wildtype, CNS WHO grade 4. However, in addition to the *MLH1* mutation, the high frequency of *BCORL1* alteration among many other alterations is distinct from the usual GBM. Methylation profiling was not possible given the limited tissue. Saliva-based Ambry panel testing confirmed a germline *MLH1* c.589-2A>G pathogenic mutation.

Adjuvant radiation therapy to 40 Gy in 15 fractions was initiated but discontinued at 24 Gy due to wound dehiscence and hematoma as a result of cachexia and anticoagulation for DVT. A wound washout and re-closure were performed without complication. Given MSI-high status, MMR deficiency, and elevated TMB, the decision was made to proceed with pembrolizumab 200 mg every 3 weeks.

## Discussion

Spinal GBM can be diagnostically challenging. Surgical biopsy carries substantial risk of neurological morbidity due to the infiltrative nature of GBM and the eloquent anatomy of the spinal cord. Moreover, limited tissue frequently restricts molecular characterization. Conventional minimally invasive diagnostic modalities, including magnetic resonance imaging (MRI) and CSF cytology, are suboptimal for detecting GBM, underscoring the need for more adequate diagnostic tools.

To our knowledge, this is the first reported case of a provisionally diagnosed spinal GBM utilizing CSF ctDNA. Our case corroborates the established low sensitivity of conventional diagnostics, including CSF cytology (6), which failed to detect malignant cells on two different samples. CSF ctDNA offers a minimally invasive approach for early diagnosis and treatment guidance, eliminating neurosurgical risk. Moreover, unmethylated *MGMT* results from Belay Vantage™ and Tempus™ informed the omission of TMZ, while detection of MSI, high TMB, and DNA mismatch repair alterations suggested Lynch syndrome and guided the decision for immunotherapy.

Although high ctDNA levels are typically observed in GBM with Ki-67 >20% (7), CSF ctDNA analysis was successful even at a lower proliferative rate (Ki-67=5%-10%) in our case. Belay Summit™ showed superior concordance with the Tempus™ assay on the tumor compared to GTC Liquid Trace™, with presurgical CSF ctDNA findings of *PTEN* and *MLH1*/*MSH2* mutations, which were suggestive of GBM and Lynch syndrome. While *PTEN* is not a diagnostic criterion for GBM, *PTEN* mutations are found in 30-40% of GBMs (8) and raised the suspicion for GBM. Our case illustrates the complementary potential diagnostic value of CSF ctDNA, demonstrating its capacity to identify genomic alterations not detected by tissue-based NGS in the diagnosis of GBM. A low-level *EGFR* gain was identified in our patient’s tumor only by GTC Liquid Trace™ and not by Belay Summit™ or Tempus™. *EGFR* amplification is present in ~50% of GBMs and constitutes a diagnostic criterion for GBM (9). This finding raises the possibility of either a false positive or a subclonal event missed by tissue sampling due to low VAF (10). Moreover, ctDNA analyses may include non-tumor-derived alterations, which predominantly occurs after cytotoxic therapy. In our case, Belay Summit™ was performed prior to cyclophosphamide and GTC Liquid Trace™ was performed after cyclophosphamide.

Although notable discrepancies were observed between Belay Summit™ and GTC Liquid Trace™, this case was not intended as a head-to-head analytical comparison of these two platforms. A critical confounder is the difference in sampling timepoints. Belay Summit™ was performed pre-chemotherapy, whereas both GTC Liquid Trace™ and tumor tissue profiling via Tempus™ were obtained following cyclophosphamide and TMZ. It remains unclear whether chemotherapy-induced cytotoxicity affected the performance characteristics of GTC Liquid Trace™, potentially through treatment-related alterations resulting in ctDNA shedding or fragmentation. While cytotoxic therapy may theoretically increase ctDNA release through tumor cell lysis, it may also reduce overall tumor burden or alter the integrity of the DNA, thereby impacting the sensitivity of the assay. Furthermore, our patient’s tumor was *MGMT* unmethylated, with decreased response to alkylating agents and he only received 3 days of TMZ. Lastly, though GTC Liquid Trace™ and Tempus™ were performed at the same treatment timepoint, discordance between these two assays was observed. This discordance may reflect low or absent ctDNA shedding from specific tumor subclones.

Another discordance was observed with the *TP53* mutation (VAF 1.9%) detected by Belay Summit™ but absent in the matched tumor. This discrepancy may reflect the technical limitations of tissue NGS. GBMs are heterogeneous tumors, and tissue NGS relies upon the sample of tissue provided to and selected by the neuro-pathologist for processing. Conventional NGS is constrained by error rates that limit reliable detection of variants at ≤1% VAF noise (11), whereas ctDNA integrates signal from spatially and genetically distinct tumor subclones. Consistent with this, Pan et al. demonstrated that CSF ctDNA can identify mutations not detected in matched tumor specimens in brainstem gliomas (4). Taken together, these findings support a key advantage of ctDNA in its ability to capture low-frequency and spatially heterogeneous alterations that may be missed by single-site tissue biopsy (4, 12, 13). Lastly, the discordant TMB (not detected on Belay Summit™ and high TMB observed on Tempus™) may be representative of the ongoing genomic evolution of our patient’s tumor under selective pressure given that Belay Summit™ was performed early in his disease course and Tempus™ was performed later in his disease course.

Concordant alterations were also observed. A *BRCA1* mutation was identified in both CSF and tumor. *BRCA1* dysfunction contributes to genomic instability and therapeutic resistance in GBM, with potential implications for treatment with PARP inhibition. Similarly, *CDKN2A/B* homozygous deletion, a common GBM alteration, was detected by Belay Summit™ and Tempus™, with variable VAFs, reflecting the differences between CSF ctDNA and tissue sampling profiling. Collectively, this case highlights that CSF ctDNA may complement tissue NGS by uncovering clinically relevant mutations missed due to sampling bias and may provide a more comprehensive view of GBM genomic heterogeneity.

Concordance between CSF ctDNA and tissue NGS in GBM is not well established, with limited paired tissue data. Our case adds to this limited body of knowledge and emphasizes the potential of for CSF ctDNA in CNS tumors to enable timely, precision-guided care. CSF ctDNA represents a promising tool for the diagnosis of CNS tumors, particularly in cases where lesions are difficult or high-risk for biopsy (14). CSF ctDNA carries future promise to guide targeted therapies, longitudinally monitoring tumor evolution, and improving real-time diagnostic accuracy.

## Limitations

We acknowledge limitations in this study. First, a single case is not generalizable to all spinal glioblastomas predisposed by Lynch syndrome. Second, assessment of the longitudinal response to immunotherapy in our patient is constrained by the limited duration of follow-up. Third, the CSF assays were collected at different timepoints during our patient’s disease course and the disparate results may reflect differences in disease burden and the ongoing genetic evolution of GBM. Lastly, potential differences in specimen volumes, storage, and timing of processing may have also attributed to the mismatch between the test results.

## Conclusion

Cerebrospinal fluid circulating tumor DNA is a promising ancillary tool to enable patient-specific genomic stratification and targeted therapy in difficult-to-biopsy cases. We report a case of a 37-year-old man in whom prospective CSF ctDNA analysis (Belay Summit™ and GTC Liquid Trace™) supported a provisional spinal GBM diagnosis with suspected Lynch syndrome. Paired tumor-based sequencing (Tempus™) confirmed glioblastoma, and germline testing (Ambry™) established Lynch syndrome, thereby guiding therapy for immunotherapy.

## Data Availability

The original contributions presented in the study are included in the article/supplementary material. Further inquiries can be directed to the corresponding author.
